# Pressure-Sensitive Paint: Effect of Substrate

**DOI:** 10.3390/s111211649

**Published:** 2011-12-14

**Authors:** Mark Kenneth Quinn, Leichao Yang, Konstantinos Kontis

**Affiliations:** Aero-Physics Laboratory, George Begg Building, University of Manchester, Sackville Street, Manchester, M13 9PL, UK; E-Mails: leichao.yang@postgrad.manchester.ac.uk (L.Y.); k.kontis@manchester.ac.uk (K.K.)

**Keywords:** pressure-sensitive paint, PSP, AA-PSP, TLC, substrate, sensitivity, photodegradation

## Abstract

There are numerous ways in which pressure-sensitive paint can be applied to a surface. The choice of substrate and application method can greatly affect the results obtained. The current study examines the different methods of applying pressure-sensitive paint to a surface. One polymer-based and two porous substrates (anodized aluminum and thin-layer chromatography plates) are investigated and compared for luminescent output, pressure sensitivity, temperature sensitivity and photodegradation. Two luminophores [tris-Bathophenanthroline Ruthenium(II) Perchlorate and Platinum-tetrakis (pentafluorophenyl) Porphyrin] will also be compared in all three of the substrates. The results show the applicability of the different substrates and luminophores to different testing environments.

## 1. Introduction

Pressure-sensitive paint (PSP) is a method of optically measuring the absolute spatial pressure distribution over an entire surface. Compared to traditional point measurement techniques, this has the advantage of giving significantly more information about the flow physics and can allow for integration of pressure, giving forces and moments on aerodynamic surfaces [1], visualizing and quantifying shock structures [2,3] and even estimating altitude in flight [4]. The theory and working principles of PSP have been documented by several researchers [5,6] and will only be briefly recounted here.

PSP is based on the mechanism of oxygen quenching which involves the non-radiative deactivation of an excited photo-active molecule (luminophore). A luminophore is excited to an electronic state higher than its ground state by absorbing light of a specific wavelength. This excited luminophore can return to its ground state by either a radiative or non-radiative process. Radiative processes, which involve the emission of light, include fluorescence and phosphorescence and are usually grouped together under the term luminescence. The wavelength difference between the absorbed and emitted light is known as the Stokes shift [7]. It is a preferable characteristic that PSP luminophores have a large Stokes shift to allow the signals to be separated easily. Non-radiative processes include internal conversion to a different electronic state and then the release of heat, or external conversion via contact with an external molecule, in this case oxygen. Oxygen is an extremely good quenching molecule, as it has an unusual electronic ground state which is easily excited [8].

**Figure 1.**
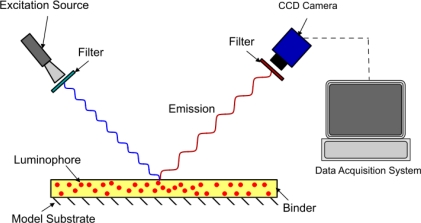
Polymer based PSP.

**Figure 2.**
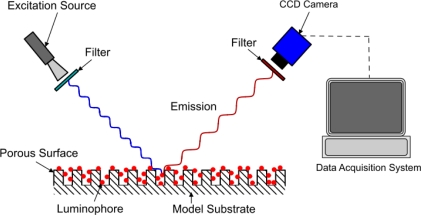
Porous based PSP.

The method of modeling oxygen quenching depends on the method of application of the PSP; however, the outcome is largely the same. There are two conventional methods of applying PSP: polymer- and porous-based (see Figures 1 and 2). Traditional polymer-based PSP involves suspending the luminophore in an oxygen-permeable polymer layer. Henry’s law states that, at constant temperature, the amount of gas dissolved in a solid is proportional to the pressure of the gas at the surface. This can be combined with Dalton’s law of partial pressures to relate the dissolved oxygen concentration, and therefore the level of quenching, to the pressure acting on the surface. Porous-based PSP involves adsorbing the luminophore onto a highly porous surface. The two most common porous PSP substrates are anodized aluminum (AA) and thin layer chromatography (TLC) plates. An anodized aluminum substrate requires that the model be made of a high-grade aluminum alloy or pure aluminum, whereas TLC plates can be used in an off-the-shelf fashion and applied directly to any flat surface. The luminophore molecules are directly exposed to the test gas, therefore porous-based PSP has a much faster response time when compared with its polymer counterpart [9], as oxygen diffusion through a polymer layer requires a finite time. However, as the luminophores are so exposed to the test gas, they are very easily quenched, leading to a very low luminescent output, even with low concentrations of oxygen. As outlined by Sakaue and Sullivan [9], porous PSP is deactivated by one of two methods: adsorption/surface diffusion or target collision. These two methods become more or less prevalent depending on the surface pressure and the surface temperature.

Typical response times for the various methods of application can be seen in Table 1. The thickness of the polymer layer has a significant effect on both the response time and the emission signal level [15]. The values presented in Table 1 are of similar thickness for ease of comparison, however if the thickness is reduced the response times can be reduced by an order or magnitude [16]. The pore size and depth found on anodized aluminum has a strong effect on the response of AA-PSP, this is discussed in detail by Sakaue [11] and Gregory *et al.* [6].

The methods of application shown here require that a reference image is taken at a known reference pressure. This allows for the elimination of effects such as non-uniform illumination or a spatial variation in luminophore concentration. Regardless of the method of application of PSP, the relationship between intensity and pressure is given by the Stern-Volmer equation [Equation (1)]. The non-linearity of this equation for different substrates was shown by Gregory and Sullivan [17].

(1) $$\frac{I_{\mathrm{ref}}}{I}=A\left(T\right)+B\left(T\right){\left(\frac{P}{P_{\mathrm{ref}}}\right)}^{\gamma }$$

where *I* is the intensity, *P* is the pressure and *γ* is an empirical parameter from the Freundlich isotherm and is normally equal to one for polymer-based PSP (polymer-based PSP does exhibit some non-linear behavior near vacuum conditions). Unfortunately, coefficients *A*(*T*) and *B*(*T*) are functions of temperature. This gives rise to what is (usually) the largest source of error in applications of PSP: the temperature-induced error. It should be noted that the coefficients *A*(*T*) and *B*(*T*) do not have the same formulations for polymer- and porous-based PSP; however, they are commonly represented in this way.

Depending on the nature of the application, it may be favorable to use a different substrate. It is the aim of this study to show the applicability of the different application methods for two commonly used luminophores: tris-Bathophenanthroline Ruthenium(II) Perchlorate [Ru(dpp)_3_] and Platinum-tetrakis (pentafluorophenyl) Porphyrin (PtTFPP). If a fast response time is required, then porous substrates are clearly the only type viable [10]. However, in steady flow conditions, response time is not as important, and the sensitivity and signal output are much more important.

This paper will present the signal output, pressure sensitivity, temperature sensitivity and photodegradation characteristics for anodized aluminum, TLC plates and polymer-based samples of Ru(dpp)_3_ and PtTFPP luminophores to show their applicability to different flows and experiments.

## 2. Sample Preparation

**Figure 3.**
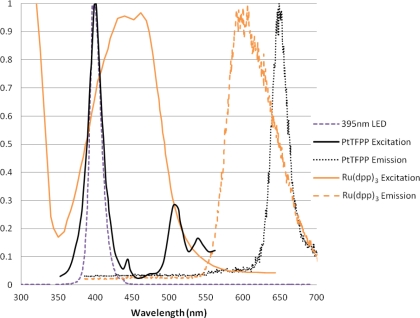
Illumination Source and PSP Spectra.

The two luminophores used in this experiment are very commonly used for a variety of applications. Both of these luminophores have a large Stokes shift, and therefore give signals which are easily separable from the incident light. Figure 3 shows the absorption and emission spectra of these two luminophores when excited with 395 nm LED lamps (these will be described later). The emission signal was measured using the Princeton Instruments ICCD spectrometer with a 530 nm long pass filter.

### 2.1. Polymer-Based

The polymer-based samples [referred to here as *Ru(dpp)*_3_ *Polymer* and *PtTFPP Polymer*] were prepared on 10 × 10 mm square aluminum plates. These plates were sprayed with 3 base coats of Ambersil matt white acrylic paint Ral9010, to give a uniform reflective surface on which to apply the PSP. This base coat was sanded using P1200 grade abrasive paper to ensure a smooth surface finish. This base coat also acts as a reflector, reflecting the unabsorbed incident light back through the polymer layer and also reflecting the emission towards the imaging device. The polymer paint itself is an in-house formulation consisting of Methyl triethoxysilane (MTEOS) as the sol-gel binder, as it is known to have excellent oxygen permeability and good adhesion [18], with ethanol and HCl as solvents. The luminophore molecules were dissolved in a combination of the solvents to the same concentration of approximately 4 mM *±* 0.2 mM. The paint was applied to the prepared base coat in 16 light coats using a modeler’s airbrush. A smooth, fast sweeping action was used to ensure uniformity and each coat was allowed to dry before application of subsequent coats. The preparation of the samples took place in as dark an environment as possible so as not to photodegrade the paint during the application process. Immediately after the PSP had been applied, the sample was cured in an oven at 343 K for 7 hours.

### 2.2. TLC Plate

Thin layer chromatography (TLC) plates have been used by several researchers as a cost-effective porous substrate for porous PSP [6,19]. Baron *et al.* [12] showed that the frequency response of such a combination could approach 100 KHz. Silica gel TLC plates can be used as a porous substrate, as luminophore molecules are known to adsorb onto the surface [20]. TLC plates have the advantage that they are white and, therefore, possess the same reflecting characteristics as the polymer base coat. Unfortunately, TLC plates are extremely fragile and can only be used on very simple and flat geometries. The TLC samples [referred to here as *Ru(dpp)*_3_ *TLC* and *PtTFPP TLC*] were prepared by dipping 10 × 10 mm squares of HPTLC silica gel 60 from MERCK Chemicals International in a 0.3 mM solution of the luminophore dissolved in dichloromethane. These values were chosen as they were shown to have the greatest sensitivity and signal output [21,22]. The TLC samples were allowed to soak in the luminophore solutions for 5 minutes before being allowed to dry in a dark environment.

### 2.3. Anodized Aluminum

The anodized aluminum plates [referred to here as *Ru(dpp)*_3_ *AA* and *PtTFPP AA*] were prepared by using high-grade aluminum alloy as anodes and cathodes in a 1 molar solution of phosphoric acid with a 20 V potential difference across them. The anodization process at the anode was left for 30 minutes before post-treatment. The voltage and anodization time are the defining parameters of pore depth and width and are explored in the review paper by Gregory *et al.* [6]. The anodized aluminum was then cut into two small squares (approximately 10 × 10 mm) which were soaked in the same luminophore solutions as mentioned previously for 5 minutes each and dried in a similar fashion.

PtTFPP has been applied to a porous substrate by Sakaue *et al.* [23]. In their work, Sakaue *et al.* used a higher concentration (1 mM) of luminophore when dipping the anodized aluminum as PtTFPP is not easily adsorbed onto a surface and, according to the Langmuir isotherm, the amount of luminophore adsorbed onto a surface is dependent on the concentration of the solution. In this study it was decided to stay consistent between the two luminophores and make solutions in the same concentration of 0.3 mM. However, this led to an extremely small amount of PtTFPP being adsorbed.

## 3. Experimental Setup

**Figure 4.**
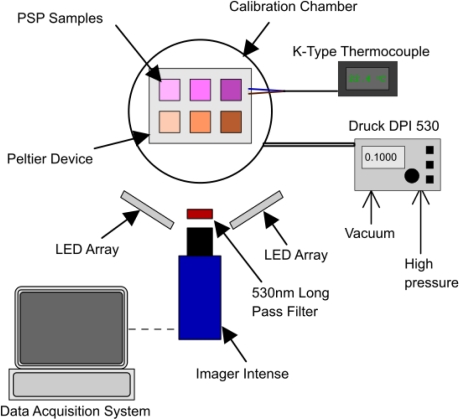
Schematic of the experimental setup.

All six of the PSP samples were mounted simultaneously in a sealed chamber with a quartz window for optical access. The pressure in the chamber was varied from a vacuum up to 2 bar in 0.1 bar intervals, using a Druck DPI 530 pressure controller which has an accuracy of approximately 0.001 bar. Over this pressure range, the temperature was maintained constant by using a Peltier thermo-electric device and was monitored using a K-type thermocouple. A schematic of the experimental setup is shown in Figure 4. The top row of samples is PtTFPP, the bottom row is Ru(dpp)_3_ and, from left to right, the columns are AA, TLC and polymer.

The samples were excited using two in-house built LED lamps, each consisting of 192, UV5TZ-395-30 LEDs manufactured by Bivar which have a peak wavelength of 395 nm and a full-width half-maximum of 20 nm. The LEDs were operated using a current limiting power supply to maintain constant current for all of the tests conducted. The emission signal of the LED lamps was measured using the Princeton Instruments ICCD Spectrometer and did not give any detectable signal below 330 nm or above 420 nm up to near IR. LED lamps make excellent PSP excitation sources as they are monochromatic and avoid the complications of filter combinations shown by Gongora-Orozco *et al.* [24]. The imaging device used was the LaVision Imager Intense 12 bit CCD camera with a 530 nm long pass filter, in conjunction with an IR cut-off filter, both purchased from Edmund Optics. The transmission of the filter combination varies from 97% at 610 nm to 94% at 650 nm. The Imager Intense has a quantum efficiency of 45% at 610 nm and 35% at 650 nm, with a maximum frame rate of 10 Hz. In order to make use of the full-well capacity of the CCD (18,000 e*^−^*), the exposure time was set at 7.5 ms. The images were stored on a Windows-based PC, processed using Davis 7 and ImageJ software.

Apart from the photodegradation tests, the LED lamps were switched off when the pressure was being varied so as to minimize the photodegradation effect.

### 3.1. Image Processing

The signal gathered by CCD imaging devices can be improved by averaging multiple images in order to reduce the photon shot noise [25]. In this test 50 images were averaged in order to create one image. These images were then passed through a 5 × 5 linear filter to help reduce spatial noise. The images were then stacked in ImageJ and intensity data was extracted. The intensity was measured over an area of approximately 7 × 7 mm through the stack for each of the samples present in the image.

## 4. Results

The following section shows the results of this study and discusses the implications on the applicability of the substrate and luminophore combination. The PtTFPP AA sample consistently appears to have the lowest sensitivity to any of the tests. This is likely to be due to the low amount of luminophore adsorbed on to the substrate. For this reason the PtTFPP results will not be discussed at length but will still be presented for completeness.

### 4.1. Intensity Output

**Figure 5.**
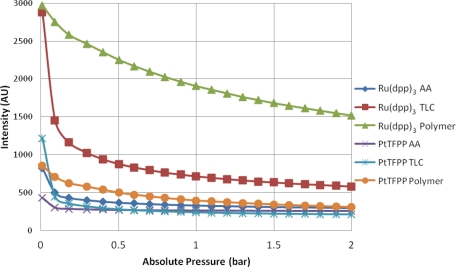
Intensity Output *vs.* Pressure.

Figure 5 shows the intensity output of the 6 different samples as the pressure is varied from approximately zero up to 2 bar. As expected, the main trend is for the intensity output to decrease as the amount of gas (specifically oxygen) in the chamber is increased. All of the samples exhibit some degree of non-linearity between the absolute pressure in the chamber and the intensity output. This is especially true at low pressures. The polymer-based PSP samples give the strongest luminescent output at almost every test condition. This is likely due to the concentration of luminophore that is present on the surface. It is almost impossible to guarantee that there is the same mass of luminophore adsorbed onto the porous surfaces as there is on the sprayed sample. A surprising result is that at the vacuum condition, PtTFPP TLC actually gives a stronger output than PtTFPP Polymer.

For all of the substrates tested, the Ru(dpp)_3_ samples gives a higher output than their PtTFPP counterparts. This is because Ru(dpp)_3_ has a higher quantum yield than PtTFPP [26], meaning it gives more output for a given input intensity. This is well illustrated in Figure 5.

**Figure 6.**
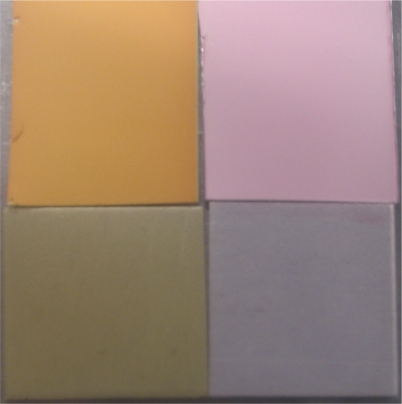
Comparison of Porous Samples. Clockwise from top right: PtTFPP TLC, PtTFPP AA, Ru(dpp)_3_ AA, Ru(dpp)_3_ TLC.

The TLC samples give very high signal levels at the vacuum condition, significantly higher than the AA samples, but shows a similar level of non-linearity. This could likely be because the TLC surface is a stronger adsorbent than the AA surface, and therefore more of the luminophore is present on the surface (see Figure 6 for a comparison under regular white light).

The AA samples show very low signal output for both luminophores and appear almost completely quenched at 0.3 bar. A possible reason for this is that the anodized surface is matt grey in appearance and is therefore not as reflective as the polymer base coat and the TLC surface. Therefore, some of the emitted light may be lost. The Ru(dpp)_3_ AA sample shows a higher signal output than the PtTFPP AA sample. This is likely due to a combination of two factors:

1. PtTFPP gives a lower signal output as mentioned before;
2. The PtTFPP was not adsorbed well onto the AA surface, as it shows only a very faint color compared to the Ru(dpp)_3_ AA sample.

### 4.2. Pressure Sensitivity

**Figure 7.**
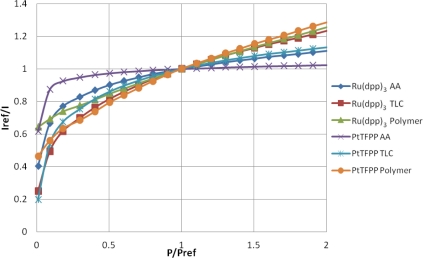
Stern-Volmer Plot.

The well-known Stern-Volmer plot for all of the samples is shown in Figure 7, where the reference condition is ambient pressure. This figure shows the normalized intensity output against the normalized pressure ratio and is known as the Stern–Volmer plot. The gradient of the lines shown in Figure 7 represent the pressure sensitivity of the PSP.

**Figure 8.**
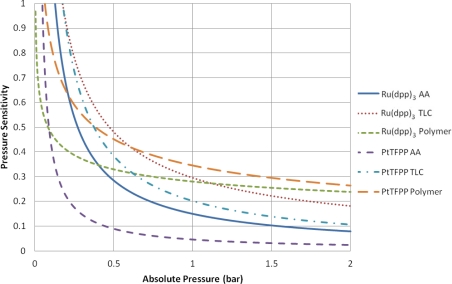
Pressure Sensitivity *vs.* Absolute Pressure.

Table 2 contains a series of modified Freundlich models which fit the data shown in Figure 7. To illustrate the variable pressure sensitivity of each sample, the derivative of the functions shown in Table 2 are shown in Figure 8. The pressure sensitivity is given by Equation (2):

(2) $$\mathrm{PS}=\frac{d\left(\frac{I_{\mathrm{ref}}}{I}\right)}{d\left(\frac{P}{P_{\mathrm{ref}}}\right)}$$

The PtTFPP Polymer sample showed the highest pressure sensitivity above 0.65 bar absolute pressure, despite its low signal output seen in Figure 5. Both polymer-based PSP samples exhibit good pressure sensitivity across the pressure range tested and showed a reasonably linear response in the high pressure region. This is to be expected from Henry’s law.

The TLC samples show extremely high pressure sensitivity across the lower pressure ratios. These samples exhibit a very non-linear sensitivity across sub-atmospheric pressures and almost linear sensitivity across higher than atmospheric pressures. This highly non-linear behavior is expected to be a result of the two quenching mechanisms present. However, at higher than atmospheric pressures the Ru(dpp)_3_ TLC sample shows almost the same level of sensitivity as the Ru(dpp)_3_ Polymer sample. The same is not strictly true of the PtTFPP TLC sample; however, it does exhibit significantly higher pressure sensitivity than the PtTFPP AA sample over the same range.

The AA samples also show extremely high pressure sensitivity in the vacuum region; however, these samples level off much sooner and exhibit poor pressure sensitivity towards high pressures. This is most evident for the PtTFPP AA sample, which exhibits an 5.8% change in intensity ratio for 5 fold change in pressure ratio. The reason for the low pressure sensitivity of the PtTFPP AA sample is likely to be due to the low level of adsorbed luminophore on the surface. However, to a lesser extent, the same low sensitivity is true of the Ru(dpp)_3_ AA sample, which exhibits a 23.1% intensity ratio change for a 5 fold increase in pressure ratio. This low pressure sensitivity means that AA PSP is only really applicable under conditions where the local concentration of oxygen is very low, such as in the hypersonic wind tunnel at the University of Manchester [27,28], or in tests using Nitrogen [29]. These results show that over a wide pressure range, a linear calibration curve *cannot* be used.

The low-speed application of pressure-sensitive paint is a topic which has gathered much interest over the past years. Low-speed flow exhibits small pressure changes and most low-speed facilities are *open loop*, meaning that they exhaust to atmospheric pressure. As PSP is an absolute pressure sensor, low-speed applications are extremely challenging, as the change in pressure ratio can often be $\Delta \frac{P}{P_{\mathrm{ref}}}\approx 0.01$ or even lower. Over such a small range of pressure changes, it is reasonable to expect that the pressure sensitivity is linear. Clearly the highest pressure sensitivity is the most favorable for low-speed measurements. Around ambient pressure. the PtTFPP Polymer sample shows the highest pressure sensitivity, followed closely by the Ru(dpp)_3_ TLC and Ru(dpp)_3_ Polymer samples. Quinn *et al.* [30] showed the challenges associated with applying PSP to such small pressure changes and compared the PtTFPP Polymer and Ru(dpp)_3_ Polymer paints on a full-field PSP test. Their results showed that PtTFPP Polymer gives a lower degree of spatial noise than Ru(dpp)_3_ Polymer and as such could be tested at lower velocities.

### 4.3. Temperature Sensitivity

**Figure 9.**
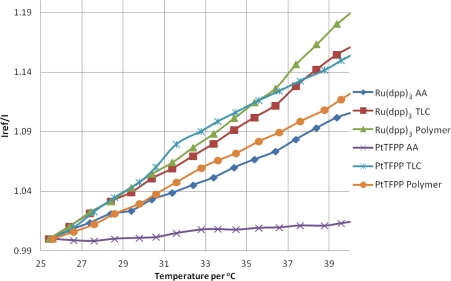
Temperature Sensitivity of PSP Samples.

The temperature sensitivity of PSP can be broken down into two components: the intrinsic temperature dependency of the luminophore and the temperature dependent oxygen permeability of the binder/substrate material. Figure 9 shows the response of the PSP samples at atmospheric pressure with increasing temperature. As the temperature is increased, the luminophores are deactivated more readily and therefore give a lower signal than the reference condition, meaning an increase in intensity ratio. The overall intensity decrease per degree is given in Table 3. The average value temperature sensitivity of the luminophores and substrates here is approximately ≈−0.9% °C^−1^.

### 4.4. Photodegradation

**Figure 10.**
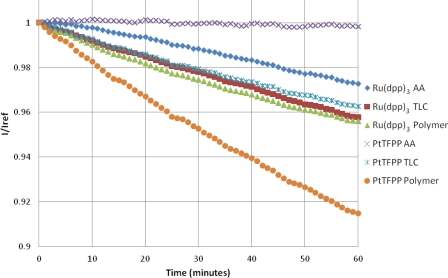
Photodegradation of PSP Samples.

The photodegradation of porous PSP was studied by Egami and Asai [31]. They showed that the photodegradation of porous PSP can be extremely severe, and, depending on the luminophore, can be up to 47% per hour. Figure 10 shows that, in this study, the highest photodegradation rate is seen with the PtTFPP Polymer sample at approximately 8.9% per hour. The photodegradation rate for Ru(dpp)_3_ Polymer paint is approximately half that of the PtTFPP Polymer sample. A linear fit of the photodegradation is given in Table 3.

## 5. Conclusions

It has been shown from this study that tris-Bathophenanthroline Ruthenium(II) Perchlorate has a higher quantum yield than Platinum-tetrakis (pentafluorophenyl) Porphyrin for the same substrate type. This is especially noticeable when comparing polymer-based samples, where the Ru(dpp)_3_ Polymer sample has a quantum yield between 3 and 5 times higher than the PtTFPP Polymer sample. Therefore, if signal level is critical, it is favorable to use a Ruthenium complex as the luminophore.

It is easier to hold a large amount of luminophore in a polymer binder than it is to adsorb a large amount onto a porous surface. This leads to the polymer-based samples having higher signal output levels than the other substrate types. However, the response time of polymer-based paint means that it is only really applicable to steady flow measurements. If unsteady measurements are required then porous substrates need to be used.

Pressure sensitivity (especially for porous substrates) varies wildly depending on the amount of oxygen present. The PtTFPP Polymer sample has the highest pressure sensitivity across the largest range. This makes the PtTFPP Polymer paint the most applicable for low-speed measurements. However, if the oxygen concentration is low (either very low working pressures or with a nitrogen-controlled atmosphere) AA and TLC samples have better sensitivity. TLC samples exhibit better sensitivity than AA samples over a wider range, regardless of luminophore. It would appear that TLC plates are the best all-round performer. The disadvantage of TLC plates is that they are extremely brittle and can only be applied to extremely simple geometries. Porous substrates are also easily contaminated and can be extremely affected by changes in local humidity due to their hydrophilic nature.

Photodegradation is an unfortunate and unavoidable side effect of the oxygen quenching process. In this study photodegradation results have been presented that show that polymer-based PSP samples suffer from this more than porous samples. The photodegradation characteristics of PtTFPP are much more dependent on the substrate than Ru(dpp)_3_.

The choice of substrate for PSP tests can seriously affect performance, both in terms of pressure and temporal resolution. It is the hope of the authors that this article simplifies the choice for the reader by highlighting the advantages and disadvantages of the commonly used types.

## Figures and Tables

**Table 1.** Typical PSP response times.

Luminophore	Substrate\application method	Response time	Reference
Ru(dpp)_3_	Anodized aluminum	50 *μ*s	Asai *et al.* [10]
Ru(dpp)_3_	Anodized aluminum	43 *μ*s	Sakaue [11]
Ru(dpp)_3_	Anodized aluminum	50 *μ*s	Asai *et al.* [10]
PtTFPP	Anodized aluminum	57 *μ*s	Sakaue [11]
H_2_TFPP	TLC plate	25 *μ*s	Baron *et al.* [12]
Ru(dpp)_3_	TLC plate	70 *μ*s	Sakaue *et al.* [9]
Ru(dpp)_3_	TLC plate	O 10 *μ*s	Sakamura *et al.* [13]
PtOEP	Polymer	0.82 s	Carroll *et al.* [14]
Ru(dpp)_3_	Polymer	0.48 s	Carroll *et al.* [14]

**Table 2.** Pressure Sensitivity.

Sample	Power Fit Relationship
Ru(dpp)_3_ AA	$\frac{I_{\mathrm{ref}}}{I}=-1.08041+2.08086{\frac{P}{P_{\mathrm{ref}}}}^{0.07182}$
Ru(dpp)_3_ TLC	$\frac{I_{\mathrm{ref}}}{I}=0.00567+0.99448{\frac{P}{P_{\mathrm{ref}}}}^{0.29692}$
Ru(dpp)_3_ Polymer	$\frac{I_{\mathrm{ref}}}{I}=0.63428+0.36356{\frac{P}{P_{\mathrm{ref}}}}^{0.76952}$
PtTFPP AA	$\frac{I_{\mathrm{ref}}}{I}=0.9976{\left(\frac{P}{P_{\mathrm{ref}}}-0.00904\right)}^{0.04508}$
PtTFPP TLC	$\frac{I_{\mathrm{ref}}}{I}=-1.68405+2.67791{\frac{P}{P_{\mathrm{ref}}}}^{0.07548}$
PtTFPP Polymer	$\frac{I_{\mathrm{ref}}}{I}=0.42754+0.56332{\frac{P}{P_{\mathrm{ref}}}}^{0.61116}$

**Table 3.** Temperature Dependency and Photodegradation.

Sample	Intensity decrease % per °C	Photodegradation Rate % per hour
Ru(dpp)_3_ AA	−0.64	−2.7
Ru(dpp)_3_ TLC	−0.93	−4.1
Ru(dpp)_3_ Polymer	−1.07	−4.4
PtTFPP AA	−0.10	−0.2
PtTFPP TLC	−0.89	−4.1
PtTFPP Polymer	−0.75	−8.5
